# Validity of a simple Internet-based outcome-prediction tool in patients with total hip replacement: a pilot study

**DOI:** 10.1177/1357633X13519040

**Published:** 2014-04

**Authors:** Cornel Stöckli, Robert Theiler, Eduard Sidelnikov, Maria Balsiger, Stephen M Ferrari, Beatus Buchzig, Kurt Uehlinger, Christoph Riniker, Heike A Bischoff-Ferrari

**Affiliations:** 1 Department of Rheumatology, Triemli City Hospital, Zurich, Switzerland; 2 Centre on Ageing and Mobility, University of Zurich and City Hospital Waid, Zurich, Switzerland; 3 Department of Orthopaedic Surgery, Triemli City Hospital, Switzerland; 4 Geriatric Clinic, University Hospital Zurich, Switzerland

## Abstract

We developed a user-friendly Internet-based tool for patients undergoing total hip replacement (THR) due to osteoarthritis to predict their pain and function after surgery. In the first step, the key questions were identified by statistical modelling in a data set of 375 patients undergoing THR. Based on multiple regression, we identified the two most predictive WOMAC questions for pain and the three most predictive WOMAC questions for functional outcome, while controlling for comorbidity, body mass index, age, gender and specific comorbidities relevant to the outcome. In the second step, a pilot study was performed to validate the resulting tool against the full WOMAC questionnaire among 108 patients undergoing THR. The mean difference between observed (WOMAC) and model-predicted value was −1.1 points (95% confidence interval, CI −3.8, 1.5) for pain and −2.5 points (95% CI −5.3, 0.3) for function. The model-predicted value was within 20% of the observed value in 48% of cases for pain and in 57% of cases for function. The tool demonstrated moderate validity, but performed weakly for patients with extreme levels of pain and extreme functional limitations at 3 months post surgery. This may have been partly due to early complications after surgery. However, the outcome-prediction tool may be useful in helping patients to become better informed about the realistic outcome of their THR.

## Introduction

Osteoarthritis (OA) is the leading cause of disability in old age.^1^ Despite its frequency, OA is a condition that is poorly understood, and specific treatments to prevent or reverse the condition are lacking. Pain associated with OA can be relieved with analgesics, and, later, with total joint replacement. However, like any surgical procedure, THR is associated with some morbidity and mortality^2^, and does not fully alleviate pain or restore function in most individuals^3^. This may be partly explained by psychosocial and comorbid conditions that affect the outcome independent of the procedure.^4^

Pain and functional limitation of the joint are the two factors that bother patients the most and cause them to undergo THR. The WOMAC (Western Ontario and McMaster Universities) osteoarthritis index is the most widely used tool that measures these outcomes. The WOMAC questionnaire has been thoroughly validated in clinical trials in patients with hip and knee OA.^5,6,7^ Its length of 24 items, however, restricts its use in elderly people.^8^ A further limitation of the WOMAC is that key demographic variables and co-morbid conditions, described as independent predictors of outcome in the literature, are not considered. For example, body mass index, mental health, comorbidities and age are important independent predictors of the outcome after THR.^9,10,11^

The aim of the present study was to develop and validate a simple and comprehensive Internet-based assessment tool to help middle-aged and elderly patients undergoing THR predict their pain and functional level after the surgery.

## Methods

In the first step, the key questions were identified by statistical modelling in a data set of 375 patients undergoing THR. In the second step, a pilot study was performed to validate the resulting tool against the full WOMAC questionnaire among 108 patients undergoing THR. The study was approved by the appropriate ethics committee.

### Development of the outcome calculator

In developing the hip QUALITOUCH outcome calculator (QOC),^12^ we used data that were originally collected for a Swiss THR study.^13^ There were 375 participants, of mean age 70 years (SD 11), 261 (70%) of the participants were age 65 years or older, and 187 (50%) were men.

A predictive model was developed to estimate the change in WOMAC scores for pain as a result of THR surgery. A second predictive model was developed to estimate the change in WOMAC function scores. We identified the WOMAC questions that were the strongest predictors of change in WOMAC pain and function scores from pre- to postoperative value. To keep the tool short, we limited it to the two most predictive WOMAC questions that assessed pain and the three most predictive questions that evaluated function by regression analyses. The appropriate questions were identified by regression: the change in pain was regressed on WOMAC questions 1–5, and the change in function was regressed on WOMAC questions 8–24. Potential explanatory variables were removed by backwards elimination.^14^ A standard package was used (SAS 9.2, SAS Institute Inc., Cary, NC, USA)

The initial pain model contained scores for all pain-related WOMAC questions. The question with the highest *P*-value (as long as *P* > 0.05) was eliminated from the model; then the model was re-run and the procedure repeated until two questions with the smallest *P*-values remained in the model. These questions were: “How intense is your joint pain walking on a flat surface?” and “How intense is your joint pain sitting or lying in bed?”.

Using the same procedure, the following three questions were selected for the post-surgery function model: “How difficult is it to go shopping?”, “How difficult is it to put on your socks?” and “How difficult is it sitting on a toilet and standing up again?”.

At the second stage of model development, 17 comorbidity indicators: 13 from the Sangha comorbidity index (high blood pressure, heart disease, stroke, depression, diabetes, obesity, cancer, drug abuse, lung diseases, kidney diseases, liver diseases, stomach diseases, anaemia) plus four additional yes/no questions assessing musculoskeletal pain (back pain, prior total hip replacement, total knee replacement, chronic pain), age, gender and body mass index, were included in the predictive models and exposed to a forward stepwise selection procedure. We included the four additional questions on musculoskeletal pain based on the work by Nilsdotter and colleagues^15^ showing that hip OA patients with musculoskeletal comorbidities, such as low back pain and OA of the non-operated hip, have less long term functional improvement after THR. The procedure was carried out separately for the pain and the function predictive models, and all variables maintained in the models were required to achieve a *P*-value of 0.15 or less. The following conditions were significant for prediction of pain: kidney disease, liver disease, back surgery, obesity, high blood pressure, total hip replacement, use of alcohol or drugs and total number of medicines taken by the patient. The following conditions were significant for prediction of function: back surgery, back pain, obesity, high blood pressure, total hip replacement, lung disease, height, use of alcohol or drugs and total number of medicines taken by the patient.

The final models predicted change between pre- and post-surgery levels of pain and function. The 95% confidence intervals (95% CI) calculated by the models were very wide: on average 80 WOMAC points for pain and 72 points for function. Since the entire WOMAC scale is 100 points, such wide confidence intervals are of limited value. As a reasonable compromise between precision and usability, we used 70% confidence intervals which were on average 42 and 38 points for pain and function respectively.

### Validation of the outcome calculator

All participants answered the hip outcome calculator questions before surgery and an electronic version of the WOMAC questionnaire (version 3.1) before and at 3 months after THR surgery. We chose the 3 month follow-up as the time where most improvement after surgery is usually achieved. The range of the WOMAC subscale score was transformed to 0 to100 points, where a score of 0 indicated no pain or best function and 100 indicated maximum pain or absolute loss of function.

Patients’ responses to the WOMAC questionnaire at 3 month follow-up and the outcome calculator before surgery were evaluated and compared to test the validity of the latter. The outcome calculator gave three estimates for change in pain and function: an average predicted change, and lower and upper limits for the 70% confidence interval as worst case and best case scenario estimates (see above on the reason why 70% rather than 95% CI were applied).

The predictive model calculates probable change in pain and function. The change is calculated as a negative value in WOMAC score units, say -20. Then this calculated change is subtracted from the pre-surgery WOMAC score reported by a patient. The resulting difference is the predicted post-surgery score.

Post-surgical WOMAC pain and WOMAC function scores reported by the participants were used as comparisons to evaluate validity of the model predictions.

### Statistical analysis

The final model-predicted point estimates and calculated 70% confidence intervals for change in pain and function used the standard procedure for the least-squares linear model.

For the validity test of the outcome calculator, we calculated the differences between predicted post-surgical pain and function levels and the actual observed WOMAC pain and function levels reported by the study participants at 3 months after surgery. Average differences between observed and predicted values were calculated to evaluate size and direction of bias. Model performance was further assessed by estimating the probability that the predictive model under- or overestimated the true value and the probability that model-predicted results would not deviate from the true value by more than 20% on either side.

To assess the intra-rater reliability of the outcome calculator, we evaluated agreement between two assessments in 11 participants by calculating the intraclass correlation coefficient (ICC) for the results for pain and function.

## Results

The characteristics of the participants enrolled in the validation study are summarised in Table 1. The average pre-surgery WOMAC scores for pain and function were 48.0 (SD 18.0) and 50.7 (SD 18.2) points respectively.

**Table 1.** Characteristics of the validation sample (n = 108).

Number (%)
Men	54 (50)
Women	54 (50)
Mean age, y (SD)	72.9 (8.5)
Age ≥65 y	89 (82)
Mean body mass index, kg/m^2^ (SD)	27.9 (4.9)
Number with at least one comorbidity	89 (82)
Comorbidities
High blood pressure	57 (53)
Obesity	42 (39)
Heart disease	26 (24)
Back pain	25 (23)
Chronic pain	15 (14)
Stroke	12 (11)
Diabetes	10 (9)
Lung disease	6 (6)
Kidney disease	4 (4)
Back surgery	4 (4)
Depression	3 (3)
Cancer	2 (2)
Mean WOMAC pain score (SD)	48.0 (18.0)
Mean WOMAC function score (SD)	50.7 (18.2)

### Validity

On average, the predictive models tended to be conservative and to overestimate postoperative pain levels by 1.12 WOMAC points (95% CI −3.76 to +1.52) and function levels by 2.49 points (95% CI −5.27 to + 0.29), see Figures 1 and 2. Eighty five per cent of model-predicted pain levels and 82% of function levels were no further than 15 WOMAC points from the true values reported by the participants.

**Figure 1A.**
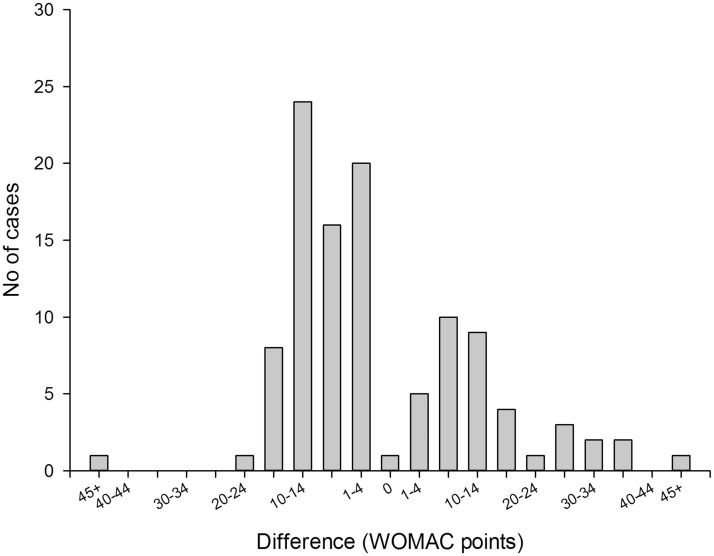
Differences between observed and model-predicted values of pain. The model overestimated the observed values (gave conservative results) in 71 of 108 patients (68%). In 25 patients (23% of the study population) the model underestimated the observed pain levels by 10–14 WOMAC points.

**Figure 1B.**
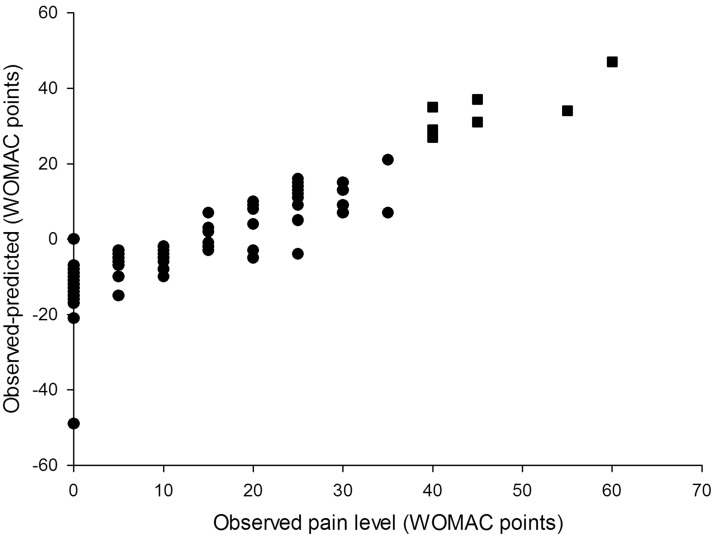
Differences between observed and model-predicted pain levels. The model performed best (achieved smallest differences between observed and predicted values) in the low and medium range of WOMAC pain scale: between 10 and 40 WOMAC points. In all cases when the model predicted confidence intervals did not include reported values, the patients reported either no pain or high levels of pain (square symbols).

The distribution of differences between observed WOMAC scores at 3 months after surgery and model-predicted values for pain and function models (before surgery) are shown in Figures 1A and 2A respectively. Both models tended to be conservative and often (56% of the time for both models) overestimated actual pain or functional impairment by 1–14 points on the 100-point WOMAC scale.

Overall, the models for pain and function demonstrated similar predictive performance (Figures 1B and 2B). Both models tended to overestimate extremely low levels of pain and functional impairment (between 0 and 10 points on the WOMAC scale) and underestimate high levels (greater than 40 points). The degree of underestimation increased, on average, as observed levels of pain and function became higher. In our sample there were no participants who reported levels of pain or function above 65 WOMAC points, so we could not test the performance of our models in that range of values. As a result, the usability of the tool for patients with extreme levels of pain and functional limitation (>60 WOMAC points) may be limited.

The model-predicted estimates were within 20% of the observed value 48% of the time for the pain model and 57% of the time for the function model (Table 2). The 70% CI included observed pain level values 92% of the time and function level values 91% of the time. In cases when the model-predicted confidence intervals did not contain observed values (9 for pain level and 10 for function), observed pain levels were at the extreme ends of the spectrum in the regions where the models performed relatively weakly. Six out of 8 observed pain level values that were modelled and not covered by the predicted 70% confidence interval were the six highest pain level values in the entire study population and ranged from 40 to 60 WOMAC points (Figure 1B). The same was true for 8 out of 10 observed function level values that were outside the predicted confidence interval (Figure 2B). The 95% CI included observed pain and function levels 98% and 96% of the time respectively (Table 2). However, the CI were on average 80 WOMAC points wide for pain and 72 points wide for function measurements, which severely limited their use as a tool for providing information in best and worst cases. For this reason only the 70% CI was used in the outcome calculator.

**Figure 2A.**
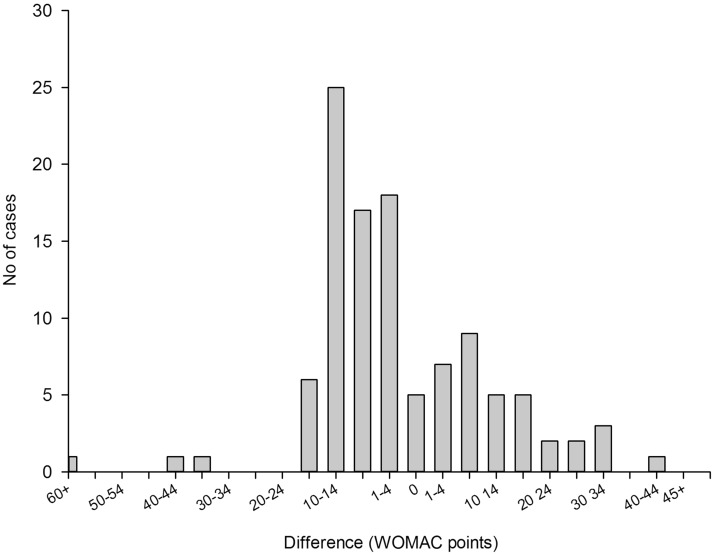
Differences between observed and model predicted values of function level. The model overestimated the observed values (gave conservative results) in 70 of 108 patients (65%). In 25 patients (23% of the study population) the model underestimated the observed function levels by 10–14 WOMAC points.

**Figure 2B.**
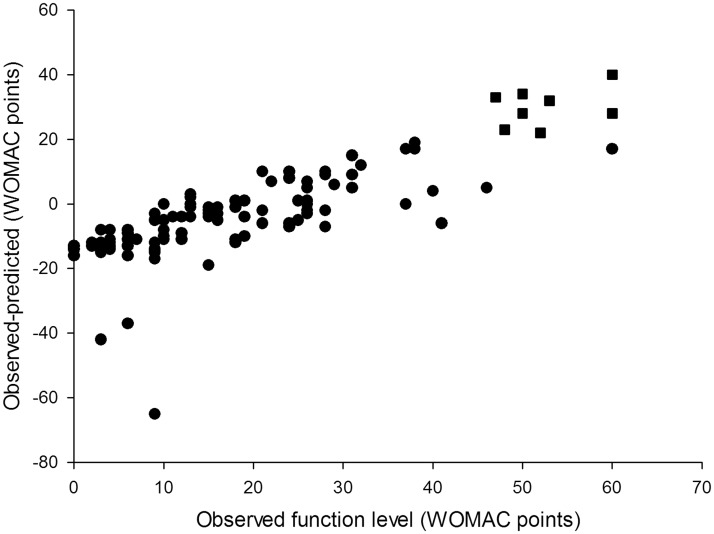
Differences between observed and model-predicted function levels. The model performed best (achieved smallest differences between observed and predicted values) in the low and medium range of WOMAC function scale: between 10 and 40 WOMAC points. In 8 out of 10 cases when the model predicted confidence intervals did not include reported values, the patients reported high levels of function loss (square symbols).

**Table 2.** Model-predicted point estimates and reported pain and function levels.

Number of cases	**%**
**Pain level prediction**
Point estimate
Model underestimated for more than 20%	21	19
Prediction within 20% of observed value	52	48
Model overestimated for more than 20%	35	32
95% confidence interval
Includes observed value	106	98
Does not include observed value	2	2
70% confidence interval
Includes observed value	99	92
Does not include observed value	9	8
**Function level prediction**
Point estimate
Model underestimated for more than 20%	14	13
Prediction within 20% of observed value	62	57
Model overestimated for more than 20%	32	30
95% confidence interval
Includes observed value	104	96
Does not include observed value	4	4
70% confidence interval
Includes observed value	98	91
Does not include observed value	10	9

More than 50% of the study participants had various post-operative complications, the most frequent of which was post-operative haematoma. Participants whose level of pain was predicted with no more than 20% error were 13% less likely to have post-surgical complications after THR. Among patients whose level of functional impairment was predicted with an accuracy of within 20%, the likelihood of post-surgical complications was 9% less than for participants whose functional impairment levels was predicted less accurately. These differences, however, were not significant. Patients whose observed levels of pain and function were not covered by the model-predicted confidence intervals also tended to have higher probability of post-surgical complications by 14% and 17% respectively. These differences were not significant.

Intra-rater reliability between two pain and function measurements repeated by 11 patients seven days apart was high. The ICC was identical for both pain and function measurements: ICC = 0.84 (95% CI 0.55–0.95).

## Discussion

The hip outcome calculator was designed to be a user-friendly, self-applied and Internet-based tool for patients undergoing THR to predict their pain and functional levels after surgery. In a pilot study, we found that it performed moderately well at predicting pain and function 3 months after THR. The models predicted post-surgical levels of pain and function with moderate accuracy. The models tended to provide conservative predictions which slightly overestimated observed pain and functional impairment. In a recent study, Mahomed and colleagues reported that patient expectation is an important predictor of outcome after THR.^16^ Our tool may help by providing patients with realistic expectations about the outcomes of their THR surgery. However, the hip outcome calculator will need further study and validation. Its overall performance was weak at high levels of pain and functional impairment. This may have been due to the models being designed with a limited number of questions so that the tool remained simple and user friendly. It may also have been due to the datasets that were used to develop and validate the models containing few data on extremely severe levels of pain and functional impairment after THR.

In summary, the hip outcome calculator demonstrated moderate validity, but performed weakly for patients with extreme levels of pain and extreme functional limitations at 3 months post surgery. This may in part be explained by early complications after surgery. However, the outcome calculator may be useful in helping patients to become better informed about the realistic outcome of their THR.
